# New Approaches in Oncology for Repositioning Drugs: The Case of PDE5 Inhibitor Sildenafil

**DOI:** 10.3389/fonc.2021.627229

**Published:** 2021-02-26

**Authors:** Marian Cruz-Burgos, Alberto Losada-Garcia, Carlos D. Cruz-Hernández, Sergio A. Cortés-Ramírez, Ignacio Camacho-Arroyo, Vanessa Gonzalez-Covarrubias, Miguel Morales-Pacheco, Samantha I. Trujillo-Bornios, Mauricio Rodríguez-Dorantes

**Affiliations:** ^1^ Laboratorio de Oncogenómica, Instituto Nacional de Medicina Genómica, Ciudad de México, Mexico; ^2^ Unidad de Investigación en Reproducción Humana, Instituto Nacional de Perinatología-Facultad de Química, Universidad Nacional Autónoma de México, Ciudad de México, Mexico; ^3^ Laboratorio de Farmacogenómica, Instituto Nacional de Medicina Genómica, Ciudad de México, Mexico

**Keywords:** drug repurposing, sildenafil, PDE5 inhibitors, cGMP, cancer, therapy

## Abstract

The use of already-approved drugs to treat new or alternative diseases has proved to be beneficial in medicine, because it reduces both drug development costs and timelines. Most drugs can be used to treat different illnesses, due their mechanisms of action are not restricted to one molecular target, organ or illness. Diverging from its original intent offers an opportunity to repurpose previously approved drugs to treat other ailments. This is the case of sildenafil (Viagra), a phosphodiesterase-5 (PDE5) inhibitor, which was originally designed to treat systemic hypertension and angina but is currently commercialized as erectile dysfunction treatment. Sildenafil, tadalafil, and vardenafil are PDE5 inhibitors and potent vasodilators, that extend the physiological effects of nitric oxide and cyclic guanosine monophosphate (cGMP) signaling. Although most of the biological implications of these signaling regulations remain unknown, they offer a large therapeutic potential for several diseases. In addition, some PDE5 inhibitors’ molecular effects seem to play a key role in different illnesses such as kidney disease, diabetes mellitus, and cancer. In this review, we discuss the molecular effects of PDE5 inhibitors and their therapeutic repurposing in different types of cancer.

## Introduction

The development of new drugs is a long, arduous and expensive process; thus, drug repurposing represents an alternative and effective strategy, which reduces both development costs and timeliness by using already approved compounds to offer alternative clinical options.

Drug repurposing, or repositioning, is defined as the process of finding new medical indications and uses for existing drugs (1, 2). The available studies of bioavailability, pharmacokinetics, dosage, safety, efficacy, and toxicity for already approved drugs, are the keystone for drug repositioning. This data can be translated into savings in time and capital, which represents a new hope to develop low-cost therapies for different diseases like cancer (3). In other words, drug repurposing contributes to implementing new treatments and new target discovery in the shortest time possible and at an affordable cost (4).

The main examples of drug repurposing success are multiple myeloma and colorectal cancer treatment. Thalidomide was initially developed as a potent sedative and became popular among pregnant women for relieving morning sickness. After thalidomide was associated with congenital malformations, it was withdrawn from the market. But in 2006, was officially approved to be used in myeloma treatment in combination with the steroid dexamethasone (5). Thalidomide (100–200 mg) has shown antiangiogenic, pro-apoptotic and immunomodulatory effects, while dexamethasone enhances such effects (20–40 mg) (6). In the case of colorectal cancer, low doses of aspirin (75–300 mg) proved to reduce the risk of invasion and metastasis in Duke stages B (muscle invasion), C (lymph node invasion) and D (metastasis), after 5 years of therapy (7).

On the other hand, some drugs that are still found in clinical trials with potential as repositioned drugs. The compound AZD4017 was initially developed to treat type 2 diabetes mellitus, obesity, and metabolic syndrome, nevertheless this compound has demonstrated its effectiveness in patients with idiopathic intracranial hypertension (IIH) (400 mg AZD4017 twice daily for 12 weeks). AZD4017 effect over IIH is explained because its molecular mechanism of action; as an oral selective competitive inhibitor of the intracellular enzyme 11β-HSD, AZD4017 prevents the conversion of cortisone to active cortisol, reducing intracranial pressure (8). In early stage-surgically breast cancer the use of propanolol (80–160 mg daily), reduced intratumoral mesenchymal markers and promoted the infiltration of immune cells in phase II clinical trials (9). Originally propanolol was designed to treat hypertension, angina, anxiety, and cardiac arrhythmias because it is a non-selective adrenergic β-blocker (10).

Interestingly, phosphodiesterase 5 (PDE5) inhibitors, as sildenafil has proved to be effective in several pathologies, as pulmonary hypertension, heart failure and Alzheimer disease, but its use as an anticancer drug has emerged, considering that cancer therapies are urgently needed (11, 12).

## Sildenafil History

Many FDA registered drugs have more effects than previously reported, and their molecular mechanisms of action remain unclear. This is the case of sildenafil, commercially known as Viagra, a PDE5 inhibitor. As a vasodilator drug, sildenafil’s original purpose was to treat both hypertension and angina, but it is currently used in erectile dysfunction treatment.

In 1983, Dr. Giles Brindley studied the effects of phenoxybenzamine, an antiadrenergic with potential use for erectile dysfunction. Dr. Giles proposed muscle relaxants could produce erections (13). Muscle relaxants were known to influence both, vascular and non-vascular smooth muscle, however, the mechanism by which these drugs produced the erection was unknown and these drugs presented a major disadvantage as they had to be injected directly into the penis to produce the erection (14). For these reasons, researchers improved their efforts to characterize the molecular mechanisms of muscle relaxants in erectile dysfunction. At the time, the pharmaceutical company Pfizer started a research program to develop a selective PDE5 inhibitor to increase the nitric oxide (NO)/cyclic guanosine monophosphate (cGMP) pathway for the treatment of angina.

In 1985, Pfizer’s project leader, Dr. Simon Campbell, carried out preclinical trials for the compound UK-92-480, which was capable to induce coronary artery dilatation in patients (15, 16). During these trials, men reported an erectogenic effect with the UK-92-480. A year later, its use as an antianginal drug was ruled out and repurposed (17). But the molecular mechanisms of this effect remained unclear until 1991, when Dr. Ignarro et al., found that NO increases cGMP concentrations in smooth muscle cells of the penis (18). Also, Dr. Solomon demonstrated that NO was a second messenger of cGMP-mediated erection and also proved the presence of the NO synthase (NOS) in the penis and blocking erections with NOS inhibitors (19).

**Figure 1:**
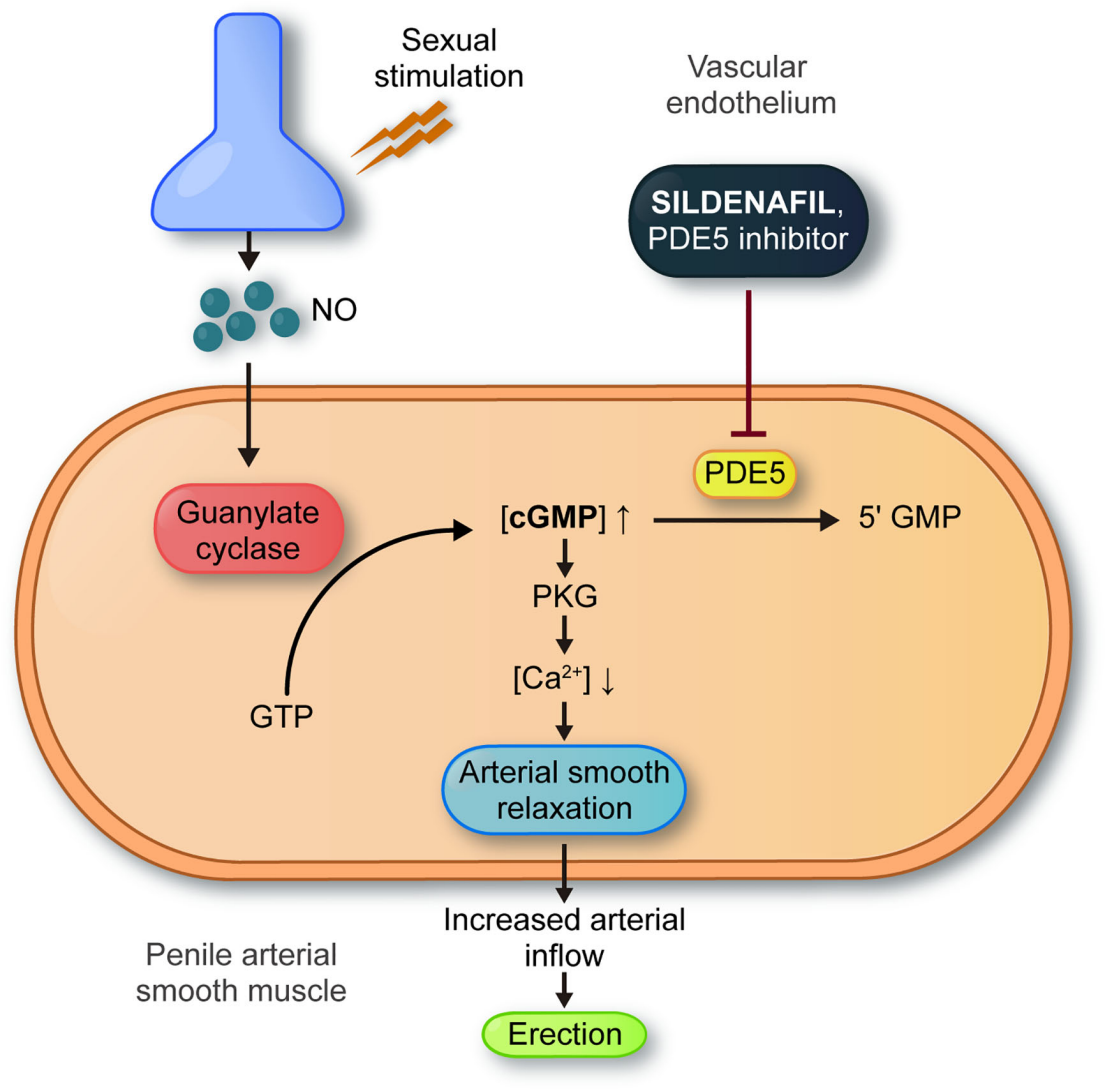
General mechanism of action of sildenafil in erectile dysfunction. Sexual stimulation results in the release of nitric oxide (NO) from nerves and endothelial cells directly into the penis. NO enters the smooth muscle cells and binds to guanylyl cyclase. This interaction results in production of 3′-5′–cyclic guanosine monophosphate (cGMP) from guanosine 5′-triphosphate (GTP). Sildenafil blocks degradation of cGMP, inhibiting PDE5. Accumulation of cGMP activates cGMP-dependent protein kinase (PKG) leading to a decrease in intracellular calcium levels. Relaxation of arterial and trabecular smooth muscle increased arterial inflow and the rigidity of penile erection.

By the mid 90’s the evidence showed the potential of sildenafil as an erectile dysfunction treatment, so Pfizer started extensive research to prove the drug specificity and elucidate its molecular mechanism of action. They found sildenafil’s molecular target was PDE5, highly expressed in the corpora cavernosa of the penis and its vasculature, but poorly in the myocardium, which provides tissue specificity to the compound. In conclusion, sildenafil’s molecular mechanism of action in the penis was completed. Sildenafil increases cGMP levels in response to NO-releasing by sexual stimulation, which results in smooth muscle relaxation and increases blood flow to the corpora cavernosa, producing an erection (**Figure 1**) (20–22).

For this reason, Pfizer’s program for treating angina shifted to treating erectile dysfunction. Sildenafil’s clinical trials for erectile dysfunction lasted from 1993 to 1998 with the FDA approval, and sildenafil was commercially released as Viagra. The same year it was approved in Europe (11). Since then, sildenafil has been the drug of choice for the treatment of erectile dysfunction (with a recommended dose of 25–100 mg) (Pfizer 2010), in addition it promoted research on human physiology and sexuality. Giving rise to the development of new PDE5 inhibitors such as vardenafil (2003), avanafil (2012), and tadalafil (2003). By 2006, research on the mechanisms of action of PDE5 inhibitors progressed leading to the identification of new applications as well as the repurposing of these drugs (23–25). In this review we will explore the potential uses of sildenafil from its use in cardiovascular diseases and erectile dysfunction to its possible utility in cancer therapy.

## Sildenafil Pharmacokinetics and Pharmacogenetics

Orally administered, sildenafil is rapidly absorbed, and it reaches a maximum plasma concentration within an hour and has a half-life of 3–4 h (the shortest among PDE5 inhibitors). Its steady state volume of distribution is 105L, exceeding the total volume of body water, which suggests drug distribution into tissues and tissue binding. It is also highly bound to plasma proteins (26, 27).

Sildenafil shows an extensive first pass metabolism with an oral bioavailability of 40%. Hepatic CYP3A4/5, CYP3A5, CYP2D6, and CYP2C19 are responsible for its biotransformation, also CYP2D6 and CYP2C19 are unique for sildenafil (27). Interestingly, pharmacokinetic parameters as plasma drug concentrations do not correlate with the drug’s efficacy, suggesting that pharmacodynamic variability should play an important role in interindividual variation for both efficacy and side effects. Drug availability in plasma can be increased by age and concomitant drug administration of inhibitors of CYP3A4/5 such as ketoconazole, erythromycin, HIV protease inhibitors, cimetidine, antacids, and grapefruit juice. In contrast, administration of CYP3A inducers such as rifampin, phenobarbital, phenytoin, and carbamazepine will decrease sildenafil’s plasma levels (27).

**Figure 2:**
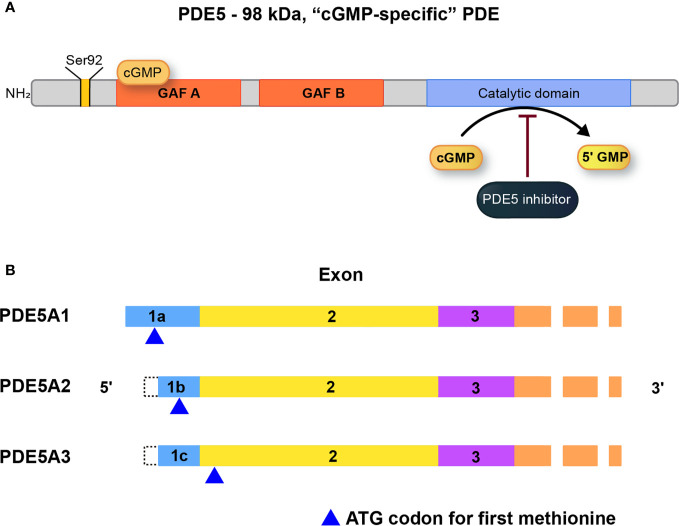
**(A)** Structure, and regulation of PDE5A. The catalytic domain is located near the C terminus of the protein and is relatively selective for cGMP as substrate at physiological levels. The substrate binding site on the catalytic domain is the target for inhibitors such as the erectile dysfunction drug, sildenafil. **(B)** PDE5A isoforms (A1, A2, A3) which differ only in the extreme 5’-end of the mRNA and accompanying amino acid sequence. For PDE5A3, the initiating codon is in exon 2, the first common exon for all the isoforms. PDE5A2 is the most widely expressed isoform [adapted from (28)].

The high affinity of cGMP binding sites of Phosphodiesterase-5 (PDE5) are now known to be on the N-terminal regulatory GAF-A (GAF domain of the enzyme which is present in cGMP-specific phosphodiesterases) (**Figure 2**). High-affinity cGMP binding only occurs to the GAF-A domain (kDa =40). Cyclic nucleotide binding to this domain is 100-fold selective for cGMP over cAMP=40 (28).

Sildenafil’s efficacy has been associated with six genetic variants of *GNB3, CYP3A4, VEGFA, ACE*, and *NOS3*, these are listed by the Pharmacogenomics Knowledge Base (PGKB), but none of them have shown a clinical utility yet (29). Variant *GNB3* rs5443 shows a higher level of confidence, indicating that the phenotypic impact of the variant in sildenafil’s efficacy is relatively strong, but not enough for clinical implementation. This variant has a frequency of 30% in Caucasians, 80% in Africans, and almost 40% in native American populations, showing an apparent population stratification which may require local dose adjustments as pharmacogenomic variants are validated with further research (30).

## Sildenafil in Different Types of Cancer

Cancer is a major public health problem worldwide, with 18,078,957 new cases and 9,555,027 deaths in 2018 cancer represents an extensive burden of disease. For these reasons, new therapies are urgently needed and drug repurposing seems an affordable option (31).

Different PDE isoenzymes have been proposed as cancer therapeutic targets, but only PDE5 high levels have been found in different types of cancer (32) therefore, suggested as targets for inhibition, potentially with an anti-cancer outcome (33). PDE5 inhibitors family includes include exisulind, sildenafil, tadalafil, vardenafil, avanafil, udenafil, while dipyridamole and cilostazol are among the non-selective drugs. Anticancer effects of PDE5 have been demonstrated in breast cancer, colorectal cancer, bladder cancer, leukemia and prostate cancer (PCa) (22).

In this section, we will discuss about the efficacy and underlying cellular mechanisms of the family of PDE5 inhibitors in the field of oncology as repositioning drugs.

### Breast Cancer

Breast cancer is the most common cancer among women (34). Even though survival rates are rising, new therapies are still needed to reduce mortality rates and increase patients’ life quality. Evidence suggests that the cGMP signaling pathway is aberrantly regulated in different cancers, including breast cancer (35–37). Some cyclic nucleotides can act as second messengers promoting protein kinase activation. In particular, cGMP activates cyclic GMP-dependent protein kinase (PKG whose activation has been recognized as an apoptotic pathway in breast cancer) (38, 39). Therefore, PDE5 inhibition promotes cGMP accumulation representing a viable strategy in breast cancer treatment. Tinsley et al., reported that inhibition of PDE5 with sulindac sulfide (SS) caused the increase of cGMP, the activation of PKG and this in turn, the inhibition in cell growth and induction of apoptosis in breast cancer cell lines (MDA -MB-231 and SK-BR-3) (40). In another study, performed by the same group, they observed that the SS and sildenafil (1µM) were potent inhibitors of cGMP hydrolysis in the MDA-MB-231 and ZR-75-1 cell lines, and these cell lines express high levels PDE5. Furthermore, they proved that PDE5 inhibition by different mechanisms, including sildenafil and taladafil treatments, diminished cell growth and induced apoptosis. These effects are explained through attenuation of β-catenin mediated transcription (41).

Recently, it was proved that treating the breast cancer cell line MDA-MB-231 with very low doses of sildenafil (25µM), impaired cancer cell proliferation, promote apoptosis and decreases tumor growth. Sildenafil treatment affects HSP90 expression, a chaperone protein which promotes degradation of PKD2, a serine threonine kinase with an important role in cancer cell proliferation and viability (42).

Karami-Terani et al. (43), demonstrated that PDE5 expression is increased in breast cancer tissues compared to normal tissue and benign tumors. Also, there is a significant correlation between PDE5 expression and tumor malignancy (44). Stimulation of pro-tumoral characteristics in stromal fibroblasts has been observed in breast cancer due PDE5 overexpression. PDE5 activates stromal fibroblasts, which produce and secrete chemokines like CXCL16, promoting cancer progression. When treating fibroblasts associated to breast cancer cells with sildenafil and vardenafil, CXCL16 levels are dropped, therefore cancer promotion is reduced. Sildenafil and vardenafil, also affect cancer stem cells (CSCs) in breast cancer. In these cells, the inhibition of PDE5 favors the accumulation of cGMP, which impedes cAMP degradation, activating PKA, and concluding in CSC elimination. This mechanism increases the efficiency of both chemotherapy and radiotherapy (45). In addition to the previously mentioned mechanisms, Baravalle et al. (46), reported that sildenafil also act as an aromatase inhibitor, a key enzyme in breast cancer progression, since it is responsible for the conversion of androgens into estrogens, thereby inducing breast cancer growth.

Due to their relaxation effects on smooth muscle and endothelial vascular permeability, PDE5 inhibitors improve blood supply to the tumor vasculature ameliorating antineoplastic drug supply in the tumor region. Greish et al. (47) analyzed the impact of sildenafil combined with doxorubicin (DOX) (an anti-cancer chemotherapy drug) in breast cancer. BALB/c mice implanted with breast cancer 4T1 cells were treated with a combination of DOX and sildenafil. The treatment showed a significant reduction in tumor size compared to those treated with doxorubicin alone. Likewise, El-Naa et al., (48) observed that sildenafil enhances the antitumor effect of cisplatin, an antineoplastic drug, in the MCF-7 cell line. Di et al. (49) also observed an increase in DOX sensitivity of MCF-7 and MDA-MB231 cells in combination with sildenafil. These authors pustulated that sildenafil decreases the side effects of conventional chemotherapeutics through its cardioprotective action. The clinical trial NCT01375699, evaluates the use of sildenafil (100 mg three times daily on the day of DOX) as a cardioprotective factor after DOX chemotherapy, in breast cancer patients. Results are still in progress but, adding sildenafil to chemotherapy has proven to be safe (50). These findings suggest that PDE5 inhibitors could be used as adjuvants in breast cancer treatment, decreasing cancer aggressiveness for breast cancer with metastatic potential. In synthesis sildenafil has protective properties against cardiovascular and side effects, caused by conventional chemotherapies.

### Prostate Cancer

It has been demonstrated that prostate cancer (PCa) cells lines, such as LNCaP, have an increased expression of PDE5 (51). Sildenafil (up to 50 µM) can sensitize cancer cells to improve the efficacy of chemotherapeutic agents. Shi et al., (52), evaluated the effect of sildenafil on ABC- transporters- mediated multi-drug resistance in cancer cells. Protein transporters ABCB1, ABCC1 and ABCG2, are responsible for pump out or extrude chemotherapeutics drugs and reduce intracellular concentration of drugs. It was observed that chemotherapeutic agents were effluxed of the cell less effectively, suggesting a role of sildenafil as a sensitizer of drug-resistant cancer cells. Moreover, the study by Das et al., (53), demonstrated that sildenafil (10 µM) improved DOX-induced apoptosis in prostate cancer cells by generating reactive oxygen species, caspase 3 and 9 upregulation, suppression of Bcl-xL and phosphorylation of Bad. In an animal model of PCa xenograft with PC3 cells in mice, the use of sildenafil significantly inhibited tumor growth by inducing apoptosis and increasing the expression of activated caspase 3. In this study, not only sildenafil was found to improve the antitumor efficacy of DOX, but it also had a protective effect against reperfusion injury/ischemia, which is a side effect induced by DOX (53). In addition, evidence in PCa points that hypoxic cell populations show an inhibition of the NO/cGMP pathway, which leads to chemoresistance and evasion of immune detection. Restoration of the NO/cGMP pathway by treatment with PDE5 inhibitors sensitizes hypoxic cells to chemotherapeutic agents (54). Similarly, sildenafil (10µM) or vardenafil but not tadalafil sensitize castration resistant PCa cells to DOX (1µM) therapy by promoting apoptosis (55).

Despite its wide therapeutic window, sildenafil may show serious cardiovascular side effects in patients with moderate to severe cardiovascular disease (56). Most studies have demonstrated that sildenafil could be useful in treating disorders after radical prostatectomy. For example, erectile dysfunction was treated with sildenafil citrate in men after radiation therapy for PCa (57). Factors like age and radiation dose are used as independent predictors to determine sildenafil efficacy after radiotherapy (58). The combination of DOX and sildenafil also ameliorated DOX-induced cardiac dysfunction. This is related to a previous study that showed an improved function of the left ventricle in PCa mice model treated with sildenafil and DOX. In other cases, PDE5 inhibitors have been evaluated as possible therapeutic agents to manage the evolution of low urinary tract symptoms (LUTS) after low dose rate brachytherapy (53). Tamsulosin and low doses of sildenafil have been applied as combination therapy. A significant improvement in the total of International Prostate Symptom Score (IPSS) but insignificant changes in the maximum flow rate (Qmax) and in the post void residual were observed. This indicates that the use of tamsulosin and low-dose sildenafil to treat LUTS following brachytherapy leads to LUTS improvement (59).The mechanism of cell death induced by the treatment with sildenafil and DOX in PCa cells involves increased surface localization of CD95 (Fas receptor or APO-is a dead receptor) in the membrane, with concomitant inactivation of NF-κB and suppression of FLIP (inhibitory protein that blocks TRAIL-mediated cell dead) and FAP-1 (Fas-associated phosphatase-1, negative regulator of Fas) expression. These mechanistic studies may contribute to expanding the use of PDE5 inhibitors in enhancing chemotherapeutic efficacy in PCa tumors expressing CD95, a surface receptor that induces apoptosis in cancer cells (60). PDE5 inhibitors have been shown to be effective in sensitizing PCa cells to antineoplastic treatments such as DOX. The sensitizing effects of PDE5 inhibitors on apoptosis induction have also been shown to affect DNA repair mechanisms (55). In other studies, the treatment with green tea and PDE5 inhibitors (vardenafil and tadalafil) in prostate cancer cells like PC-3, induce antiproliferative effects (61). Similarly, in the PC3-derived cancer stem cells (PCSC) PDE5 inhibitors (vardenafil and tadalafil) increased apoptosis in combination therapy with cisplatin (62). Other studies have evaluated the effects of vardenafil and tadalafil over proliferation after riociguat (stimulator of guanylate cyclase) treatment, indicating that PDE5 inhibitors could protect against PCa progression, however, this effect depends on the type of cells. For example, VCaP cells have high levels of cGMP synthesis and riociguat promoted proliferation in VCaP but not in LNCaP cells (63). In this line, it was observed that cells harboring TMPRSS2-ERG fusion as VCaP cells have increased risk of PCa progressions when they are treated with PDE5 inhibitors (64).

After androgen deprivation therapy a large percentage of patients who shows tumor growth are castration-resistant prostate cancer (CRPC). During this stage treatment with vincristine, an anti-mitotic and anti-microtubules agent that induces mitotic arrest and cell death mediated by caspases has been considered as a potential therapy for CRPC. However, its use is limited due to the possible neuropathies it can generate. Combining vincristine with sildenafil (10-25µM) is an approach to achieving greater efficacy at lower doses to reduce toxicity. Sildenafil has been shown to synergistically amplify the action of vincristine on tumor cells both *in vitro* (PC-3 and DU-145 cell lines) and *in vivo* (65).

Several clinical trials such as NCT00142506, NCT00544076, NCT00057759, NCT00511498 focus on the use of sildenafil as recovery therapy in erectile dysfunction after prostate cancer-related procedures, such as hormone therapy or radiation therapy and radical prostatectomy, however, there are still no clinical trials evaluating the use of sildenafil as a possible prostate anticancer drug.

### Colorectal Cancer

Colorectal cancer (CRC) is the third most common type of cancer worldwide and one of the deadliest, showing a 5-year survival rate of approximately 60% (66, 67). In addition, in recent years there has been an increase of diagnosed CRC cases in people under 50 years (68). Surgery is the first treatment option, and it is curative in 50% of the patients. Sometimes, chemotherapy or radiotherapy are given to ensure remission. Nonetheless, this surgical procedure has a high mortality rate and it is of vital importance to develop effective therapeutic strategies for the CRC treatment to ensure a positive prognosis (69, 70).

PDE5 inhibitors can reduce the incidence of intestinal cancer by altering epithelial homeostasis *via* cGMP. In rodents, treatment with azoxymethane (AOM) a carcinogenic compound and dextran sulfate sodium a polysaccharide which disrupted mucosal barrier (AOM/DSS) leads to inflammation driven to colorectal cancer. In this model, sildenafil administration protects against AOM/DSS, generated epithelial barrier dysfunction. In addition, a reduction of 50% in multiplicity of polyps was observed compared to untreated mice. Besides, polyps formed in sildenafil-treated mice showed greater differentiation, less proliferation, less inflammation, little infiltration of myeloid cells as well as a reduction in the expression of pro-inflammatory factors such as INFγ and IL-6 (71). In a study of 192,661 patients, the use of PDE5 inhibitors was shown to be associated with a decreased risk of developing colon cancer (72).

Drug combination therapy has demonstrated the potential benefits of sildenafil as an anti-tumor agent. Regorafenib is an approved drug for the treatment of colorectal cancer and hepatocellular carcinoma. Combination of regorafenib with sildenafil has been used in patients with advanced solid tumors in a safe way (73, 74). The addition of neratinib to this drug combination has a greater lethality effect on colon cancer cells. These three drugs decrease the expression of mutant K-RAS, in addition to generating a prolonged inactivation of mTOR, AKT and p70 S6K, and a reduction in markers such as PD-L1, PD-L2, ODC that increased MHCA levels, which could increase in the sensitivity of tumor cells to immunotherapies (73).

In colon cancer cell lines, apoptosis was induced using exisulind and sildenafil (75). Accelerated turnover of the intestinal epithelium shows a decrease in cGMP, which favors susceptibility to tumorigenesis. PDE5 inhibitors suppress intestinal carcinogenesis and improve epithelial barrier function, inhibiting cGPM degradation and raising its levels (76). It has been proven that sildenafil combined with curcumin increases the efficacy of 5-Fluorouracil (antineoplastic drug) in controlling CT26 colorectal tumors. The interaction of curcumin with sildenafil affects the expression of several proteins, including the protective molecule BCL-XL which decreases and increases the levels of reactive oxygen species, leading to cancer cell death. Furthermore, it was observed that the effects in the reduction of K-RAS mediated by curcumin and sildenafil were potentiated by 5-Fluorouracil (77). The clinical trial NCT03785210 evaluates if combination therapy of tadalafil (10 mg daily) with nivolumab (480 mg) and vancomycin (125 mg) could decrease liver metastasis from primary colorectal and pancreatic cancer, the study still on phase II. This suggests that sildenafil should be a powerful chemotherapeutic combined with other drugs. These approaches have demonstrated the ability of sildenafil as a potential drug to be included in first-line treatments for colorectal cancer.

### Lung Cancer

According to the most recent GLOBOCAN report, lung cancer represents 11.6% of all new cancer cases worldwide and is the leading cause of cancer-related deaths (78). Lung cancer is strongly associated with tobacco use, but it usually takes decades to develop. Therefore, a lung cancer diagnosis is rare before age 30. Social and cultural smoking patterns have led to a constant rise in lung cancer diagnosis in women, but men are still more affected. Despite treatment, survival rates are low and since the mid-1970s the increases have been minimal (79). The focus on lung cancer management should be aimed towards improving survival rates. New approaches to the treatment of cancer have been shifted towards the repurposing of drugs. moThe study of this type of drugs is reinforced by previous reports of elevated expression of PDE5 in multiple carcinomas when compared to adjacent normal tissue, including lung cancer (80, 81). The proven effect of vardenafil and tadalafil in the treatment of pulmonary hypertension boosts the anticipation for favorable results in lung cancer.

Lung cancer cells overexpress PDEs resulting in a decrease of cGMP levels, when compared to normal cells. By inhibiting their catabolism, an increase of intracellular cGMP will be observed, as a result, it may have antineoplastic effects by inhibiting tumor growth and act as an effective anticancer agent (78, 81). Recent studies have demonstrated that the combined use of platinum-based chemotherapeutic agents and PDE inhibitors have a higher antiproliferative effect on lung cancer cells than platinum monotherapy, the current standard of care (78, 82). This outcome was observed in small cell lung cancer cells, where the addition of sildenafil to the carboplatin monotherapy in small cell lung cancer cells increased apoptosis and cytotoxicity. The same effect was seen when combining carboplatin and sildenafil with either roflumilast or theophylline (78).

Moreover, when evaluating cisplatin and PDE inhibitors, only the combination with roflumilast, an inhibitor of PDE4, increased apoptosis following a 24 h incubation (78). In Non-small Cell Lung Cancer, combined therapy with PDE inhibitors showed more cytotoxicity than carboplatin monotherapy following a 48 h incubation period (78). Dipyridamole, a vasodilator, had an additive effect in the cytotoxicity of cisplatin in breast adenocarcinoma cells and non-small metastatic lung cancer cells. In contrast, dipyridamole decreased the sensitivity to cisplatin in endocervical carcinoma cells and had no effect in other studied cell lines while it also showed the same tendency when evaluating the sensitivity to the cytotoxicity of oxaliplatin. Thus, it was concluded that the effects of dipyridamole on chemo response are subject to the type of cell lines and drugs (82). Summarizing, the combination of platinum compounds with PDE inhibitors showed an increased antiproliferative effect by boosting apoptosis in lung cancer cell lines, in contrast to platinum monotherapy. Due to this collaborative effect, the combined use of these drugs has been suggested for use as an additive and maintenance treatment in lung cancer (78). The effect of dipyridamole on the cytotoxicity of doxorubicin was measured through the resistant factor, the ratio of the IC50 for doxorubicin monotherapy to that of its combination with dipyridamole (82). By means of this variable, it was concluded that 20 μM of dipyridamole enhanced the sensitivity of most cell lines to doxorubicin cytotoxicity, achieving 15-fold-increase in the sensitivity in metastatic lung cancer H1915 cells. Nonetheless, it must be noted that dipyridamole reduced the sensitivity to doxorubicin in ductal carcinoma and human papillomavirus- related cervical adenocarcinoma (82).

Another combination therapy includes sildenafil with radiotherapy in the treatment of Lewis lung carcinoma (LLC). This combination abolished the irradiation-derived immunosuppression by inhibiting expression and ARG1 activity of polymorphonuclear myeloid-derived suppressor cells (PMN-MDSC), improved the CD8+ T cells response. This shows the potential of sildenafil as an antitumor agent to delay tumor recurrence after radiotherapy in lung cancer (83).

The clinical trial NCT00752115, studies the combination therapy of sildenafil and carboplatin (cytotoxic agent) in patients with previously untreated advanced non-small-cell lung cancer. Administering 50 mg of sildenafil weekly is expected to improve the distribution and efficacy of carboplatin.

### Brain Cancer

Most cerebral lymphomas derive from the central nervous system, and their treatment includes high chemotherapy doses of methotrexate in conjunction with brain-radiation and intravenous rituximab an antibody therapy (84). A main problem for brain tumor treatment is the access through the brain blood barrier, i.e., the presence of cerebral capillary endothelium, astrocytes, pericytes, and micro vessels which hinder drug transport to the brain. PDE5 is highly expressed in the 9L glioma cell line, and in other tumor cell lines such as GL26 (mouse glioma), U87 (human primary glioblastoma) and RG2 (rat glioma).

Brain tumor models in rats have shown that the treatment with PDE5 inhibitors such as vardenafil and sildenafil in addition to DOX improves survival and reduced tumor size (85). A study in a murine model treated with an oral dose of 10 mg/kg vardenafil in brain tumor metastatic mice, increased permeability of metastatic brain tumors, without significantly affecting tight junctions of the capillary endothelium of tumors, suggesting that PDE5 inhibitors should activate its effect through stimulation of caveolae-mediated endocytosis and micropinocytosis (86). PDE5 inhibitors have also been documented to improve microvascular permeability of monoclonal antibodies such as rituximab used successfully in treatment for lymphoma (84). However, in glioblastoma multiforme (GBM), the most aggressive and lethal brain tumor, high levels of the PDE5 protein in tumor cells are associated with a less aggressive cancer, diminishing invasive potential, and DNA repair capacity of GBM cells, thus positioning PDE5 expression as an important indicator of disease prognosis. The PDE5-low and high expression groups may allow for the identification of tumors that are more invasive and resistant to radiation. This provides information that could help to predict which GBM patients will develop radiotherapy inconsistent outcomes and who might be candidates for alternative therapeutic procedures. Tumor permeability and invasive differences may be dependent on PDE5 levels offering information on the potential application of PDE5 inhibitors in GBMs PDE5 negative patients (87). Currently, the phase II clinical trial NCT01817751, tries to explain the mechanism of action of sildenafil and other drugs (sorafenib and valproic acid) in patients with recurrent high-grade glioma. Adding sildenafil to the combination therapy of sorafenib and valproic acid, may increase the concentration of sorafenib in the brain by blocking ABCG2 transporter. This could stop the growth of tumor cells by blocking enzymes essential for cell growth. In another pilot study NCT02279992, use vardenafil to increase concentration of systemic carboplatin in patients with recurrent malignant gliomas or metastatic brain cancer, using 20 mg of oral administrated vardenafil 1-h prior 100 mg carboplatin.

### Head and Neck Cancer

Head and neck tumors occur in the oral cavity, oropharynx, hypopharynx, and larynx. Most of them are histologically classified as squamous cell carcinomas (HNSCC). Different risk factors have been identified such as tobacco, excessive alcohol consumption, infections with human papillomavirus, etc., and therapeutic strategies are mainly surgery, radiotherapy, chemotherapy, or a combination of them. As it is already known, these treatments can lead to unwanted side effects that in this type of cancer can be facial disfigurement, speech problems, changes in the passage of food, among others, which significantly reduce the quality of life of the patients (88). Therefore, it is highly relevant to explore new outbreaks for the treatment of this disease.

The immune system plays a key role in the progression of HNSCC, Myeloid-derived Suppressor Cells (MDSCs) play a critical role between the innate and adaptive immune system through their ability to influence T-reg cells. In this sense, studies on the suppression of the immune response mediated by MDSC show that MDSC exploits the metabolism of L-arginine (L-Arg) to produce lymphocytes that do not respond to antigenic stimulation. Therefore, the functional elimination of MDSC can overcome the immunosuppression exerted inside tumors.

Some approaches have been made in the use of PDE5 inhibitors that can block the production of nitric oxide and arginase 1 and restore the function of tumor-specific T cells. An immunomodulatory effect has been shown by inhibiting PDE5 with tadalafil, which reduced the number of MDSC and T-reg in the blood of patients with HNSCC, while increasing the concentration of tumor-specific CD8 T cells (89). A similar study demonstrated the ability of PDE5 inhibition to increase the function of the immune system in cancer patients, confirming the observations of the Donald T. Weed group (90). *In vivo* tests in a xenograft model of athymic (nu/nu) mice inoculated with CAL27 cells, a line of squamous cell carcinoma treated with tadalafil showed a reduction in tumor weight and volume. In addition to showing that tadalafil and sildenafil reduced cell viability in a panel of HNSCC cell lines (UM1, UM6, UM47, and CAL27) (88). Two clinical trials study the effects of combined therapy with tadalafil in head & neck cancer. The clinical trial NCT02544880 use tadalafil (10–20 mg) treatment to promotes an antitumor response and creates a permissive environment that should increase the efficacy of anti-tumor MUC-1/polyICLC vaccine in patients with resectable and recurrent HNSCC. This trial proved the safety of combined treatment, which was well tolerated with no serious adverse effects or toxicities (91). The trial NCT03993353 uses combination of tadalafil (10 mg) and pembrolizumab as a safe therapy for head & neck cancer, the trial still in course.

### Chronic Lymphocytic Leukemia

Leukemias are a group of blood and bone marrow malignant diseases characterized by an increased number of leukocytes in blood and bone marrow. Leukemias are classified according to the dominantly presenting leukemia cells. In chronic lymphocytic leukemia (CLL) mature cells are affected, while in acute leukemia the main leukemia cells correspond to precursor cells. Both precursor or mature cells are altered in chronic myeloid leukemia (92).

B cells are one of the main cell types affected by lymphocytic leukemia. B cell activity depends on the production of cyclic AMP, which can be regulated by some PDE isoforms such as PDE 3,4,5, and 7. Gartner, et.al., identified that the main cyclic AMP hydrolyzing activity was performed by soluble PDE4 isoenzyme (93). PDE5 inhibitors could play an important role regulating CLL cells, sildenafil and vardenafil induce caspase-dependent apoptosis of B-CLL cells *in vitro*. For 3.5 years, chronic lymphocytic leukemia patients were treated once a week with sildenafil 50 mg, decreasing the lymphocyte count (94). In 2004 Treon (95) reported that sildenafil induced apoptosis in B cells from patients with Waldenstrom’s macroglobulinemia, an incurable B cell malignancy. Also, the role of sildenafil in combination with (−)-eEpigallocatechin-3-O-gallate (EGCG) to induce myeloid leukemia cells apoptosis, has been well described *in vitro* (61). EGCG is a polyphenol present in green tea, which is capable of inducing apoptosis in acute myeloid leukemia through acid sphingomyelinase activation. This stimulates Akt/eNOS axis upregulating vascular cGMP.

In acute myeloid leukemia (AML) a synergistic effect between sildenafil and EGCG has been described, increasing the pro-apoptotic action of EGCG. In AML PDE5 inhibitors increased cGMP concentration and apoptosis (61).

### Melanoma

Melanoma is a malignancy of the melanocytes, skin pigment producing cells and represents ~5% of all skin cancer cases. Nevertheless, it is the most lethal type of skin cancer accounting for >75% of all skin cancer deaths (96). Davies et al., reported that BRAF somatic missense mutations were present in 66% of patients with malignant melanoma, increasing BRAF kinase activity (97). Similar results were described by Alsina et al. (98). The oncogene BRAF acts through MEK and the cGMP-specific phosphodiesterase PDE5A, which enhances melanoma cell invasion by increasing cGMP and upregulating cytosolic Ca2+, contractility (99). In 2014 a prospective cohort study of 14,912 men concluded that sildenafil prescription was related to a higher risk of melanoma (100) indeed, sildenafil could promote melanoma progression.

Although there is a lot of evidence indicating melanoma relationship with the use of PDE5A inhibitors such as sildenafil, a recent meta-analysis has pointed to another possibility. Loeb, S. et al. described a meta-analysis using Medline, EMBASE and Cochrane library. They analyzed 866,049 men of whom 41,874 were diagnosed with melanoma. This study found a little association between sildenafil exposure and melanoma. They reported a significant correlation between low PDE5 inhibitors exposure with low-stage melanoma. Nevertheless, this association did not satisfy Hill’s criteria for causality (101). In synthesis more research is still needed to probe if sildenafil is associated with melanoma risk.

## Discussion

Most cancer studies that include the use of sildenafil are still under *in vitro* and *in vivo* studies. However, to the date there are different clinical trials evaluating the use of sildenafil in cancer therapy. In these trials, sildenafil is often used in similar doses to the recommended for erectile dysfunction therapy, ranging from 10 mg daily to 100 mg for at least 7 days which decreases the likelihood of overdose toxicity and demonstrates the drugs safety for this repurpose. Animal models have proved that drugs like regorafenib combined with PDE5 inhibitors decrease tumor growth. The combination of sildenafil with regorafenib decreases the expression of protective proteins such as MCL-1 and BCL-XL and the induction of death receptor signaling (73). In addition, clinical trials, like the NCT02466802 trial evaluate the use of drugs like neratinib to enhance the synergistic effect of regorafenib with sildenafil in patients with advanced solid tumors. In hepatocellular carcinoma (HCC) cytokine-induced killer cell-based immunotherapy is effective at early stages but lacks efficiency in advanced HCC. Tadalafil was used to target myeloid-derived suppressor cells (MDSC) enhancing immunotherapy effect. Results show tadalafil reduces MDSC markers and tumor size (102). Moreover, it has been found that PDE5 and PDE4 isozymes were expressed in human bladder tumor cells and were sensitive to exisulind at doses that inhibited tumor cell growth. In *in vivo* tests the treatment with exisulind was associated with a reduction in tumor size and proliferation in bladder cancer (103).

The evidence provided along the review, shows that sildenafil can be used as an anti-cancer therapy in several types of cancer; providing a tumor volume decrease, enhanced pro-apoptotic effect and a synergistic effect with chemotherapeutic agents. Nevertheless, the post-surgical distant recurrence is a common and worrying event in cancer treatment. In these cases, metastasis remains even when curative resection surgery is followed by adjuvant therapy. One of the strategies to reduce the risk of post-surgical recurrence is perioperative therapies (104), sildenafil has been studied as a possible perioperative drug. In different kinds of cancer, it is known that the increase of MDSC cells is related to poor prognosis because of its association with high grade of recurrence. PDE5 inhibitors such as sildenafil and tadalafil could affect MDSCs functions by decreasing the expression of arginase 1 (ARG1), IL4Ra, and the concentration of reactive oxygen species (ROS), which would increase the cytotoxic activity of NK cells against tumor cells (105). Currently the NCT02998736 clinical trial seeks to determine whether administration of tadalafil, before, during and after surgery can decrease the spread of abdominal cancer. In addition to tadalafil administration, the trial is looking to apply the influenza vaccine after surgery with the intention of increasing and activating NK cells. Although the trial is in phase I, it is an innovative approach to using PDE5 inhibitors such as sildenafil or tadalafil to decrease MDSCs and therefore decrease the likelihood of metastasis in patients who have already gone through a resection of primary tumors. The use of PDE5 inhibitors should be one of the great advances in drug repositioning in the area of cancer therapy.

## Concluding Remarks

**Figure 3:**
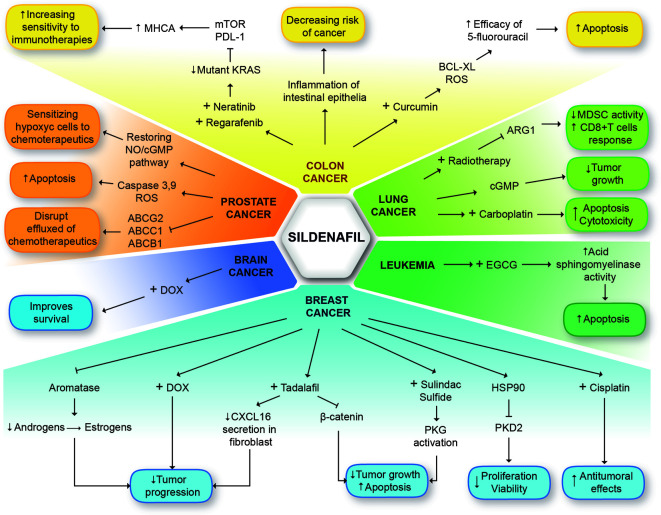
General mechanisms of sildenafil in different types of cancer. The different types of cancer are highlighted in colors. Breast cancer (light blue). Three mechanisms may decrease tumor progression: Combined therapy sildenafil + DOX, estrogen reduction by aromatase blockage or decrease secretion of CXCL16 in fibroblasts with sildenafil or tadalafil. Tadalafil can block tumor growth and promote apoptosis. The same effect is caused by combined therapy sildenafil + sulindac sulfide through PKG activation. Sildenafil can promote PKD2 degradation and decrease proliferation and viability, and enhance antitumoral effects of cisplatin. Leukemia (dark green). Sildenafil + EGCG promote activity of acid sphingomyelinase increasing apoptosis. Lung cancer (light green). Sildenafil may decrease tumor growth through cGMP and enhance cytotoxicity of carboplatin to promote apoptosis. Combined therapy of sildenafil + radiotherapy reduces activity of MSCS and promotes immune response of CD8^+^T cells. Colon cancer (yellow). Sildenafil prevents inflammation of intestinal epithelium decreasing the risk of cancer. Combined therapy sildenafil + curcumin, enhance efficacy of 5-fluorouracil to promote apoptosis. Combination of neratinib, regorafenib and sildenafil affects mutant KRAS expression to increase sensitivity of cancer cells to immunotherapies through MHCA. Prostate cancer (orange). Blocking ABC transporters. Sildenafil disrupted the efflux of chemotherapeutics promoting their activity. Sildenafil can promote caspase activity and ROS to produce apoptosis and sensitize hypoxic cancer cells to chemotherapeutics through NO/cGMP pathway restoring. Brain cancer (deep blue). Sildenafil + DOX, improves survival of cancer patients.

Drug-repurposing represents a novel strategy that could redeem time and financial resources in order to improve current therapies or to propose new ones, using already existing drugs, that benefits cancer patients widen the frame of therapeutic options. PDE5 inhibitors are a relatively new drug class, which pharmacokinetics and pharmacodynamics have not been fully elucidated. Hence, its potential for repurposing to treat other ailments is currently being explored. In this review, we delved into the evidence that supports the repositioning of PDE5 inhibitors, as anti-cancer drugs. PDE5 inhibitors can act as chemotherapeutic adjuvants, positively impacting cancer treatment. We discuss a molecular synergy between sildenafil and anticancer drugs showing encouraging outcomes for breast, lung, prostate, leukemia, head & neck, and colorectal cancer (**Figure 3**). Unfortunately, many of these synergistic effects have been observed only *in vitro* with a few clinical trials in course. Research to validate these observations *in vivo* and assess survival rates and cost-benefit of the proposed chemotherapy cooperation is still needed. Increasing repurposing of drugs such as sildenafil could improve research and cancer treatments.

Here, we summarize the mechanisms of action and diverse effects of PDE5 inhibitors, which may serve as the basis to officially suggest their repositioning to treat several types of cancer (**Table 1**). PDE5 inhibitor sildenafil has already demonstrated significant efficacy as a proapoptotic, anti-inflammatory, and immune modulator when used in combination with anticancer drugs and recently as a perioperative drug. PDE5 inhibitors offer an attractive alternative to improve current cancer chemotherapeutics. Nevertheless, more investigations are needed to demonstrate its effects and molecular mechanisms on specific types of cancer. Drug repositioning is a strategy with the potential to improve current drug efficiency that is cost-effective.

**Table 1:** Effects of PDE5 inhibitors in cancer.

Cancer type	Drug	Effect	Reference
Breast	Sulindac Sulfide	Apoptosis induction and growth inhibition	(40, 41, 47)
	Sildenafil		
	Tadalafil	Improve chemotherapy outcomes	
	Doxorubicin-Sildenafil		
Prostate	Sildenafil	Sensitizes prostate cancer cells to chemotherapy beneficial outcomes	(53, 55, 60)
Colon	Sildenafil	Decreased risk of developing colon cancer	(71–73, 76)
		Reduction in the expression of pro-inflammatory factors	
		Chemoprevention	
		Enhanced the anti-tumor efficacy of Regorafenib	
Lung	Sildenafil	Improved chemotherapy outcomes	(78)
Brain	Sildenafil	Increased tumor capillary permeability and chemotherapy synergy.	(85, 87)
	Vardenafil		
Head and Neck (HNSCC)	Sildenafil	Immunomodulatory effect increasing the function of the immune system	(88–90)
	Tadalafil		
		Reduced cell viability	
Chronic lymphocytic leukemia	Sildenafil	Apoptosis induction	(94, 95)

## Author Contributions

MC-B, SC-R, CC-H, MM-P, ST-B, and AL-G wrote sections of the manuscript. MM-P and MC-B designed figures of the manuscript and AL-G made the tables. MR-D, VG-C, IC-A, and MC-B helped supervised the project and discussed final conclusions and corrections contributed to final manuscript. MR-D, MC-B, and SC-R conceived the original idea. All authors contributed to the article and approved the submitted version.

## Funding

CONCyT grant A1-S-26446, help with funding and Instituto Nacional de Medicina Genómica give us all facilities and articles access.

## Conflict of Interest

The authors declare that the research was conducted in the absence of any commercial or financial relationships that could be construed as a potential conflict of interest.

## References

[1] Ashburn TT Thor KB. Drug repositioning: Identifying and developing new uses for existing drugs. Nat Rev Drug Discov (2004) 3:673–83. 10.1038/nrd1468 15286734

[2] Sleire L Førde-Tislevoll HE Netland IA Leiss L Skeie BS Enger PØ. Drug repurposing in cancer. Pharmacol Res (2017) 124:74–91. 10.1016/j.phrs.2017.07.013 28712971

[3] Hernandez JJ Pryszlak M Smith L Yanchus C Kurji N Shahani VM. Giving drugs a second chance: Overcoming regulatory and financial hurdles in repurposing approved drugs as cancer therapeutics. Front Oncol (2017) 7:273. 10.3389/fonc.2017.00273 29184849 PMC5694537

[4] Pantziarka P Bouche G Meheus L Sukhatme V Sukhatme VP. The Repurposing Drugs in Oncology (ReDO) Project. Ecancermedicalscience (2014) 8:442. 10.3332/ecancer.2014.442 25075216 PMC4096030

[5] Rehman W Arfons LM Lazarus HM. The rise, fall and subsequent triumph of thalidomide: lessons learned in drug development. Ther Adv Hematol (2011) 2(5):291–308. 10.1177/2040620711413165 23556097 PMC3573415

[6] Kumar S Rajkumar SV. Thalidomide and dexamethasone: therapy for multiple myeloma. Expert Rev Anticancer Ther (2005) 5(5):759–66. 10.1586/14737140.5.5.759 16221046

[7] García Rodríguez LA Soriano-Gabarró M Bromley S Lanas A Cea Soriano L. New use of low-dose aspirin and risk of colorectal cancer by stage at diagnosis: a nested case-control study in UK general practice. BMC Cancer (2017) 17(1):637. 10.1186/s12885-017-3594-9 28882113 PMC5590216

[8] Markey K Mitchell J Botfield H Ottridge RS Matthews T Krishnan A. 11β-Hydroxysteroid dehydrogenase type 1 inhibition in idiopathic intracranial hypertension: a double-blind randomized controlled trial. Brain Commun (2020) 2(1):fcz050–0. 10.1093/braincomms/fcz050 PMC7425517 32954315

[9] Hiller JG Cole SW Crone EM Byrne DJ Shackleford DM Pang JB. Pre-operative β -blockade with propranolol reduces biomarkers of metastasis in breast cancerx202F;: a Phase II randomized trial. Clin Cancer Res (2019). 26(8):1803–11 10.1158/1078-0432.CCR-19-2641 31754048

[10] Pantziarka P Bouche G Sukhatme V Meheus L Rooman I Sukhatme VP. Repurposing Drugs in Oncology (ReDO)-Propranolol as an anti-cancer agent. Ecancermedicalscience (2016) 10:680. 10.3332/ecancer.2016.680 27899953 PMC5102691

[11] Ghofrani HA Osterloh IH Grimminger F. Sildenafil: From angina to erectile dysfunction to pulmonary hypertension and beyond. Nat Rev Drug Discov (2006) 5:689–702. 10.1038/nrd2030 16883306 PMC7097805

[12] Sanders O. Sildenafil for the Treatment of Alzheimer’s Disease: A Systematic Review. J Alzheimers Dis Rep (2020) 4(1):91–106. 10.3233/ADR-200166 32467879 PMC7242821

[13] Brindley GS. Pilot experiments on the actions of drugs injected into the human corpus cavernosum penis. Br J Pharmacol (1986) 87(3):495–500. 10.1111/j.1476-5381.1986.tb10191.x 3801762 PMC1916566

[14] Gasser TC Roach RM Larsen EH Madsen PO Bruskewitz RC. Intracavernous self-injection with phentolamine and papaverine for the treatment of impotence. J Urol (1987) 137(4):678–80. 10.1016/S0022-5347(17)44172-3 3550149

[15] Jackson G Gillies H Osterloh I. Past, present, and future: A 7-year update of Viagra® (sildenafil citrate). Int J Clin Pract (2005) 59(6):680–91. 10.1111/j.1368-5031.2005.00578.x 15924597

[16] Saini JS Garg MK. VIAGRAx202F;: IS IT A WONDER DRUGx202F;? Med J Armed Forces India (2001) 57(1):44–6. 10.1016/S0377-1237(01)80090-3 PMC4925037 27365578

[17] McCullough AR. Four-year review of sildenafil citrate. Rev Urol (2002) 4:S26–S38. 16986012 PMC1476025

[18] Burnett AL. Nitric oxide in the penis - Science and therapeutic implications from erectile dysfunction to priapism. J Sex Med (2006) 3(4):578–82. 10.1111/j.1743-6109.2006.00270.x 16839312

[19] Burnett AL Lowenstein CJ Bredt DS Chang TSK Snyder SH. Nitric oxide: A physiologic mediator of penile erection. Science (80- ) (1992) 257(5068):401–3. 10.1126/science.1378650 1378650

[20] Wallis RM Corbin JD Francis SH Ellis P. Tissue distribution of phosphodiesterase families and the effects of sildenafil on tissue cyclic nucleotides, platelet function, and the contractile responses of trabeculae carneae and aortic rings in vitro. Am J Cardiol (1999) 83(5A):3C–12C. 10.1016/S0002-9149(99)00042-9 10078537

[21] Pfizer. VIAGRA (sildenafil citrate) label. Pfizer Labs (2010). https://www.accessdata.fda.gov/drugsatfda_docs/label/2010/020895s033lbl.pdf

[22] Pantziarka P Sukhatme V Crispino S Bouche G Meheus L Sukhatme VP. Repurposing drugs in oncology (ReDO)-selective PDE5 inhibitors as anti-cancer agents. Ecancermedicalscience (2018) 12:824. 10.3332/ecancer.2018.824 29743944 PMC5931815

[23] Tinel H Stelte-Ludwig B Hütter J Sandner P. Pre-clinical evidence for the use of phosphodiesterase-5 inhibitors for treating benign prostatic hyperplasia and lower urinary tract symptoms. BJU Int (2006) 98(6):1259–63. 10.1111/j.1464-410X.2006.06501.x 16956354

[24] Ferrini MG Kovanecz I Nolazco G Rajfer J Gonzalez-Cadavid NF. Effects of long-term vardenafil treatment on the development of fibrotic plaques in a rat model of Peyronie’s disease. BJU Int (2006) 97(3):625–33. 10.1111/j.1464-410X.2006.05955.x 16469038

[25] Patil CS Singh VP Kulkarni SK. Modulatory effect of sildenafil in diabetes and electroconvulsive shock-induced cognitive dysfunction in rats. Pharmacol Rep (2006) 58(3):373–80. 16845211

[26] Nichols DJ Muirhead GJ Harness JA. Pharmacokinetics of sildenafil citrate after single oral doses in healthy male subjects: Absolute bioavailability, food effects and dose proportionality. Br J Clin Pharmacol Suppl (2002) 53:5S–12S. 10.1046/j.0306-5251.2001.00027.x PMC1874258 11879254

[27] Mehrotra N Gupta M Kovar A Meibohm B. The role of pharmacokinetics and pharmacodynamics in phosphodiesterase-5 inhibitor therapy. Int J Impot Res (2007) 19(3):253–64. 10.1038/sj.ijir.3901522 16988721

[28] Kass DA Champion HC Beavo JA. Phosphodiesterase type 5: Expanding roles in cardiovascular regulation. Circ Res (2007) 101(11):1084–95. 10.1161/CIRCRESAHA.107.162511 18040025

[29] PharmGKB. Clinical Annotation for rs5443 (GNB3); sildenafil; Erectile Dysfunction (level 2B Efficacy). (2018). Available at: https://www.pharmgkb.org/clinicalAnnotation/655385125.

[30] Sperling H Eisenhardt A Virchow S Hauck E Lenk S Porst H. Sildenafil response is influenced by the G protein β3 subunit GNB3 C825T polymorphism: A pilot study. J Urol (2003) 169(3):1048–51. 10.1097/01.ju.0000058369.72348.ba 12576843

[31] Ferlay J Ervik M Lam F (IARC) IA for R on C *Cancer Today*. Available at: https://gco.iarc.fr/today/fact-sheets-cancers.

[32] Barone I Giordano C Bonofiglio D Andò S Catalano S. Phosphodiesterase type 5 and cancers: Progress and challenges. Oncotarget (2017) 8(58):99179–202. 10.18632/oncotarget.21837 PMC5716802 29228762

[33] Catalano S Panza S Augimeri G Giordano C Malivindi R Gelsomino L. Phosphodiesterase 5 (PDE5) is highly expressed in cancer-associated fibroblasts and enhances breast tumor progression. Cancers (Basel) (2019) 11(11):1740. 10.3390/cancers11111740 PMC6895904 31698786

[34] Bray F Ferlay J Soerjomataram I Siegel RL Torre LA Jemal A. Global cancer statistics 2018: GLOBOCAN estimates of incidence and mortality worldwide for 36 cancers in 185 countries. CA Cancer J Clin (2018) 68(6):394–424. 10.3322/caac.21492 30207593

[35] Li N Xi Y Tinsley HN Gurpinar E Gary BD Zhu B. Sulindac selectively inhibits colon tumor cell growth by activating the cGMP/PKG pathway to suppress wnt/β-catenin signaling. Mol Cancer Ther (2013) 12(9):1848–58. 10.1158/1535-7163.MCT-13-0048 PMC3800150 23804703

[36] Wen HC Chuu CP Chen CY Shiah SG Kung HJ King KL. Elevation of soluble guanylate cyclase suppresses proliferation and survival of human breast cancer cells. PloS One (2015) 10(4):e0125518. 10.1371/journal.pone.0125518 25928539 PMC4416047

[37] Gong L Lei Y Tan X Dong Y Luo Z Zhang D. Propranolol selectively inhibits cervical cancer cell growth by suppressing the cGMP/PKG pathway. BioMed Pharmacother (2019) 111:1243–8. 10.1016/j.biopha.2019.01.027 30841438

[38] Fallahian F Karami-Tehrani F Salami S Aghaei M. Cyclic GMP induced apoptosis via protein kinase G in oestrogen receptor-positive and -negative breast cancer cell lines. FEBS J (2011) 278(18):3360–9. 10.1111/j.1742-4658.2011.08260.x 21777390

[39] Lee K Piazza GA. The interaction between the Wnt/β-catenin signaling cascade and PKG activation in cancer. J Biomed Res (2017) 31:189–96. 10.7555/JBR.31.20160133 PMC5460607 28808213

[40] Tinsley HN Gary BD Keeton AB Zhang W Abadi AH Reynolds RC. Sulindac sulfide selectively inhibits growth and induces apoptosis of human breast tumor cells by phosphodiesterase 5 inhibition, elevation of cyclic GMP, and activation of protein kinase G. Mol Cancer Ther (2009) 8(12):3331–40. 10.1158/1535-7163.MCT-09-0758 PMC2805153 19996273

[41] Tinsley HN Gary BD Keeton AB Lu W Li Y Piazza GA. Inhibition of PDE5 by sulindac sulfide selectively induces apoptosis and attenuates oncogenic Wnt/β-catenin-mediated transcription in human breast tumor cells. Cancer Prev Res (2011) 4(8):1275–84. 10.1158/1940-6207.CAPR-11-0095 PMC3151326 21505183

[42] Chen L Liu Y Becher A Diepold K Schmid E Fehn A. Sildenafil triggers tumor lethality through altered expression of HSP90 and degradation of PKD2. Carcinogenesis (2020) 41(10):1421–31. 10.1093/carcin/bgaa001 PMC7566345 31917403

[43] Karami-Tehrani F Moeinifard M Aghaei M Atri M. Evaluation of PDE5 and PDE9 Expression in Benign and Malignant Breast Tumors. Arch Med Res (2012) 43(6):470–5. 10.1016/j.arcmed.2012.08.006 22960860

[44] Catalano S Campana A Giordano C Gyorffy B Tarallo R Rinaldi A. Expression and function of phosphodiesterase type 5 in human breast cancer cell lines and tissues: Implications for targeted therapy. Clin Cancer Res (2016) 22(9):2271–82. 10.1158/1078-0432.CCR-15-1900 26667489

[45] Klutzny S Anurin A Nicke B Regan JL Lange M Schulze L. PDE5 inhibition eliminates cancer stem cells via induction of PKA signaling. Cell Death Dis (2018) 9(2):192. 10.1038/s41419-017-0202-5 29416006 PMC5833477

[46] Baravalle R Valetti F Catucci G Gambarotta G Chiesa M Maurelli S. Effect of sildenafil on human aromatase activity: From in vitro structural analysis to catalysis and inhibition in cells. J Steroid Biochem Mol Biol (2017) 165(Pt B):438–47. 10.1016/j.jsbmb.2016.09.003 27616271

[47] Greish K Fateel M Abdelghany S Rachel N Alimoradi H Bakhiet M. Sildenafil citrate improves the delivery and anticancer activity of doxorubicin formulations in a mouse model of breast cancer. J Drug Target (2018) 26(7):610–15. 10.1080/1061186X.2017.1405427 29148852

[48] El-Naa MM Othman M Younes S. Sildenafil potentiates the antitumor activity of cisplatin by induction of apoptosis and inhibition of proliferation and angiogenesis. Drug Des Devel Ther (2016) 10:3661–72. 10.2147/DDDT.S107490 PMC5117873 27895461

[49] Di X Gennings C Bear HD Graham LJ Sheth CM White KL. Influence of the phosphodiesterase-5 inhibitor, sildenafil, on sensitivity to chemotherapy in breast tumor cells. Breast Cancer Res Treat (2010) 124(2):349–60. 10.1007/s10549-010-0765-7 20155316

[50] Poklepovic A Qu Y Dickinson M Kontos MC Kmieciak M Schultz E. Randomized study of doxorubicin-based chemotherapy regimens, with and without sildenafil, with analysis of intermediate cardiac markers. Cardiooncology (2018) 4(7):1–11. 10.1186/s40959-018-0033-2 30221011 PMC6136838

[51] Zhu B Strada SJ. The novel functions of cGMP-specific phosphodiesterase 5 and its inhibitors in carcinoma cells and pulmonary/cardiovascular vessels. Curr Top Med Chem (2007) 7(4):437–54. 10.2174/156802607779941198 17305584

[52] Shi Z Tiwari AK Shukla S Robey RW Singh S Kim I-W. Sildenafil reverses ABCB1- and ABCG2-mediated chemotherapeutic drug resistance. Cancer Res (2011) 71(8):3029–41. 10.1158/0008-5472.CAN-10-3820 PMC3078184 21402712

[53] Das A Durrant D Mitchell C Mayton E Hoke NN Salloum FN. Sildenafil increases chemotherapeutic efficacy of doxorubicin in prostate cancer and ameliorates cardiac dysfunction. Proc Natl Acad Sci USA (2010) 107(42):18201–7. 10.1073/pnas.1006965107 PMC2964209 20884855

[54] Hamilton TK Hu N Kolomitro K Bell EN Maurice DH Graham CH. Potential therapeutic applications of phosphodiesterase inhibition in prostate cancer. World J Urol (2013) 31(2):325–30. 10.1007/s00345-012-0848-7 22383129

[55] Chang JF Hsu JL Sheng YH Leu WJ Yu CC Chan SH. Phosphodiesterase type 5 (PDE5) inhibitors sensitize topoisomerase II inhibitors in killing prostate cancer through PDE5-independent impairment of HR and NHEJ DNA repair systems. Front Oncol (2019) 8:681. 10.3389/fonc.2018.00681 PMC6344441 30705876

[56] Boyce EG Umland EM. Sildenafil citrate: A therapeutic update. Clin Ther (2001) 23(1):2–23. 10.1016/S0149-2918(01)80027-8 11219477

[57] Ohebshalom M Parker M Guhring P Mulhall JP. The efficacy of sildenafil citrate following radiation therapy for prostate cancer: Temporal considerations. J Urol (2005) 174(1):258–62. 10.1097/01.ju.0000164286.47518.1e 15947650

[58] Teloken PE Parker M Mohideen N Mulhall JP. Predictors of response to sildenafil citrate following radiation therapy for prostate cancer. J Sex Med (2009) 6(4):1135–40. 10.1111/j.1743-6109.2008.01170.x 19210713

[59] Yu YD Kang MH Choi CI Shin HS Oh JJ Park DS. Clinical efficacy of combination therapy with an alpha blocker and low-dose sildenafil on post-therapy lower urinary tract symptoms after low-dose-rate brachytherapy for prostate cancer. World J Urol (2016) 34(9):1269–74. 10.1007/s00345-016-1777-7 26868648

[60] Das A Durrant D Mitchell C Dent P Batra SK Kukreja RC. Sildenafil (Viagra) sensitizes prostate cancer cells to doxorubicin-mediated apoptosis through CD95. Oncotarget (2016) 7(4):4399–413. 10.18632/oncotarget.6749 PMC4826214 26716643

[61] Kumazoe M Kim Y Bae J Takai M Murata M Suemasu Y. Phosphodiesterase 5 inhibitor acts as a potent agent sensitizing acute myeloid leukemia cells to 67-kDa laminin receptor-dependent apoptosis. FEBS Lett (2013) 587(18):3052–7. 10.1016/j.febslet.2013.07.041 23916810

[62] Liu N Mei L Fan X Tang C Ji X Hu X. Phosphodiesterase 5/protein kinase G signal governs stemness of prostate cancer stem cells through Hippo pathway. Cancer Lett (2016) 378(1):38–50. 10.1016/j.canlet.2016.05.010 27179930

[63] Hankey W Sunkel B Yuan F He H Thomas-Ahner JM Chen Z. Prostate Cancer Cell Phenotypes Remain Stable Following PDE5 Inhibition in the Clinically Relevant Range. Transl Oncol (2020) 13(9):100797. 10.1016/j.tranon.2020.100797 32454444 PMC7248418

[64] Zhou F Gao S Han D Han W Chen S Patalano S. TMPRSS2-ERG activates NO-cGMP signaling in prostate cancer cells. Oncogene (2019) 38:4397–411. 10.1038/s41388-019-0730-9 PMC6542710 30718921

[65] Hsu J-L Leu W-J Hsu L-C Ho C-H Liu S-P Guh J-H. Phosphodiesterase Type 5 Inhibitors Synergize Vincristine in Killing Castration-Resistant Prostate Cancer Through Amplifying Mitotic Arrest Signaling. Front Oncol (2020) 10:1274. 10.3389/fonc.2020.01274 32850387 PMC7427565

[66] Siegel RL Miller KD Jemal A. Cancer statistics, 2018. CA Cancer J Clin (2018) 68(1):7–30. 10.1891/9780826121646.0002 29313949

[67] NIH National Cancer Institute. Cancer Facts & Figures 2020. CA Cancer J Clin (2020) 70(1):7–30. 10.3322/caac.21590 31912902

[68] Cyhaniuk A Coombes ME. Longitudinal Adherence to Colorectal Cancer Screening Guidelines. Am J Manag Care (2016) 22(2):105–11. 26885670

[69] Bethesda M. Colon Cancer Treatment (PDQ®): Health Professional Version. In: PDQ Cancer Information Summaries. Bethesda (MD): National Cancer Institute (US); January 25 (2020).

[70] Xie YH Chen YX Fang JY. Comprehensive review of targeted therapy for colorectal cancer. Signal Transduct Target Ther (2020) 5:22. 10.1038/s41392-020-0116-z 32296018 PMC7082344

[71] Islam BN Sharman SK Hou Y Bridges AE Singh N Kim S. Sildenafil suppresses inflammation-driven colorectal cancer in mice. Cancer Prev Res (2017) 10(7):377–88. 10.1158/1538-7445.AM2017-2222 PMC5530733 28468928

[72] Sutton SS Magagnoli J Cummings TH Hardin JW. The Association Between Phosphodiesterase-5 Inhibitors and Colorectal Cancer in a National Cohort of Patients. Clin Transl Gastroenterol (2020) 11(6):e00173–3. 10.14309/ctg.0000000000000173 PMC7339197 32568473

[73] Booth L Roberts JL Rais R Cutler RE Diala I Lalani AS. Neratinib augments the lethality of [regorafenib + sildenafil]. J Cell Physiol (2019) 234(4):4874–87. 10.1002/jcp.27276 PMC6322207 30203445

[74] Poklepovic AS Gordon SW McGuire WP Thacker LR Deng X Tombes MB. Phase I study of regorafenib and sildenafil in advanced solid tumors. J Clin Oncol (2020) 38:3593. 10.1200/JCO.2020.38.15_suppl.3593 PMC11168782 38452059

[75] Thompson WJ Piazza GA Li H Liu L Fetter J Zhu B. Exisulind induction of apoptosis involves guanosine 3’,5’-cyclic monophosphate phosphodiesterase inhibition, protein kinase G activation, and attenuated β-catenin. Cancer Res (2000) 60(13):3338–42. 10910034

[76] Islam BN Browning DD. Phosphodiesterase-5 inhibitors for colon cancer chemoprevention. Aging (Albany NY) (2018) 10(9):2216–7. 10.18632/aging.101545 PMC6188491 30187889

[77] Dent P Booth L Roberts JL Poklepovic A Hancock JF. (Curcumin+sildenafil) enhances the efficacy of 5FU and anti-PD1 therapies in vivo. J Cell Physiol (2020) 235(10):6862–74. 10.1002/jcp.29580 31985048

[78] Domvri K Zarogoulidis K Zogas N Zarogoulidis P Petanidis S Porpodis K. Potential synergistic effect of phosphodiesterase inhibitors with chemotherapy in lung cancer. J Cancer (2017) 8(18):3648–56. 10.7150/jca.21783 29151951 PMC5688917

[79] Torre LA Bray F Siegel RL Ferlay J Lortet-Tieulent J Jemal A. Global cancer statistics, 2012. CA Cancer J Clin (2015) 65(2):87–108. 10.3322/caac.21262 25651787

[80] Das A Durrant D Salloum FN Xi L Kukreja RC. PDE5 inhibitors as therapeutics for heart disease, diabetes and cancer. Pharmacol Ther (2015) 147:12–21. 10.1016/j.pharmthera.2014.10.003 25444755 PMC4494657

[81] Peng T Gong J Jin Y Zhou Y Tong R Wei X. Inhibitors of phosphodiesterase as cancer therapeutics. Eur J Med Chem (2018) 150:742–56. 10.1016/j.ejmech.2018.03.046 29574203

[82] Li Q Shu Y. Pharmacological modulation of cytotoxicity and cellular uptake of anti-cancer drugs by PDE5 inhibitors in lung cancer cells. Pharm Res (2014) 31(1):86–96. 10.1007/s11095-013-1134-0 23884568 PMC3864614

[83] Zhang J Zhang L Yang Y Liu Q Ma H Huang A. Polymorphonuclear-MDSCs facilitate tumor regrowth after radiation by suppressing CD8^+^ T cells. Int J Radiat Oncol Biol Phys (2021) S0360-3016(20)3456-0. 10.1016/j.ijrobp.2020.11.038 33238192

[84] Wang R Chen W Zhang Q Liu Y Qiao X Meng K. Phosphodiesterase type 5 inhibitor Tadalafil increases Rituximab treatment efficacy in a mouse brain lymphoma model. J Neurooncol (2015) 122(1):35–42. 10.1007/s11060-014-1690-0 25524816

[85] Black KL Yin D Ong JM Hu J Konda BM Wang X. PDE5 inhibitors enhance tumor permeability and efficacy of chemotherapy in a rat brain tumor model. Brain Res (2008) 1230:290–302. 10.1016/j.brainres.2008.06.122 18674521 PMC2632551

[86] Hu J Ljubimova JY Inoue S Konda B Patil R Ding H. Phosphodiesterase type 5 inhibitors increase herceptin transport and treatment efficacy in mouse metastatic brain tumor models. PloS One (2010) 5(4):e10108–19. 10.1371/journal.pone.0010108 PMC2856671 20419092

[87] Cesarini V Martini M Vitiani LR Gravina GL Di Agostino S Graziani GE. Type 5 phosphodiesterase regulates glioblastoma multiforme aggressiveness and clinical outcome. Oncotarget (2017) 8(8):13223–39. 10.18632/oncotarget.14656 PMC5355091 28099939

[88] Tuttle TR Mierzwa ML Wells SI Fox SR Ben-Jonathan N. The cyclic GMP/protein kinase G pathway as a therapeutic target in head and neck squamous cell carcinoma. Cancer Lett (2016) 370(2):279–85. 10.1016/j.canlet.2015.10.024 PMC4711273 26551887

[89] Weed DT Vella JL Reis IM De La Fuente AC Gomez C Sargi Z. Tadalafil reduces myeloid-derived suppressor cells and regulatory t cells and promotes tumor immunity in patients with head and neck squamous cell carcinoma. Clin Cancer Res (2015) 21(1):39–48. 10.1158/1078-0432.CCR-14-1711 25320361 PMC4322895

[90] Califano JA Khan Z Noonan KA Rudraraju L Zhang Z Wang H. Tadalafil augments tumor specific immunity in patients with head and neck squamous cell carcinoma. Clin Cancer Res (2015) 21(1):30–8. 10.1158/1078-0432.CCR-14-1716 PMC4329916 25564570

[91] Weed DT Zilio S Reis IM Sargi Z Abouyared M Gomez-Fernandez CR. The Reversal of Immune Exclusion Mediated by Tadalafil and an Anti-tumor Vaccine Also Induces PDL1 Upregulation in Recurrent Head and Neck Squamous Cell Carcinoma: Interim Analysis of a Phase I Clinical Trial. Front Immunol (2019) 10:1206. 10.3389/fimmu.2019.01206 31214178 PMC6554471

[92] Juliusson G Hough R. Leukemia. Prog Tumor Res (2016) 43:87–100. 10.1159/000447076 27595359

[93] Gantner F Götz C Gekeler V Schudt C Wendel A Hatzelmann A. Phosphodiesterase profile of human B lymphocytes from normal and atopic donors and the effects of PDE inhibition on B cell proliferation. Br J Pharmacol (1998) 123(6):1031–8. 10.1038/sj.bjp.0701688 PMC1565253 9559883

[94] Sarfati M Mateo V Baudet S Rubio M Fernandez C Davi F. Sildenafil and vardenafil, types 5 and 6 phosphodiesterase inhibitors, induce caspase-dependent apoptosis of B-chronic lymphocytic leukemia cells. Blood (2003) 101(1):265–9. 10.1182/blood-2002-01-0075 12393651

[95] Treon SP Tournilhac O Branagan AR Hunter Z Xu L Hatjiharissi E. Clinical responses to sildenafil in Waldenstrom’s macroglobulinemia. Clin Lymphoma (2004) 5(3):205–7. 10.3816/CLM.2004.n.029 15636699

[96] Rebecca VW Somasundaram R Herlyn M. Pre-clinical modeling of cutaneous melanoma. Nat Commun (2020) 11(1):2858. 10.1038/s41467-020-15546-9 32504051 PMC7275051

[97] Davies H Bignell GR Cox C Stephens P Edkins S Clegg S. Mutations of the BRAF gene in human cancer. Nature (2002) 417:6892. 10.1038/nature00766 12068308

[98] Alsina J Gorsk DH Germino FJ Shih W Lu S-E Zhang Z-G. Detection of Mutations in the Mitogen-Activated Protein Kinase Pathway in Human Melanoma. Clin Cancer Res (2003) 9(17):6419–25. 14695143

[99] Arozarena I Sanchez-Laorden B Packer L Hidalgo-Carcedo C Hayward R Viros A. Oncogenic BRAF induces melanoma cell invasion by downregulating the cGMP-specific phosphodiesterase PDE5A. Cancer Cell (2011) 19(1):45–57. 10.1016/j.ccr.2010.10.029 21215707

[100] Li WQ Qureshi AA Robinson KC Han J. Sildenafil use and increased risk of incident melanoma in US men: A prospective cohort study. JAMA Intern Med (2014) 174(6):964–70. 10.1001/jamainternmed.2014.594 PMC4178948 24710960

[101] Loeb S Ventimiglia E Salonia A Folkvaljon Y Stattin P. Meta-Analysis of the Association between Phosphodiesterase Inhibitors (PDE5Is) and Risk of Melanoma. J Natl Cancer Inst (2017) 109(8). 10.1093/jnci/djx086 PMC5437700 29117385

[102] Yu SJ Ma C Heinrich B Brown ZJ Sandhu M Zhang Q. Targeting the crosstalk between cytokine-induced killer cells and myeloid-derived suppressor cells in hepatocellular carcinoma. J Hepatol (2019) 70(3):449–57. 10.1016/j.jhep.2018.10.040 PMC6380944 30414862

[103] Piazza GA Thompson WJ Pamukcu R Alila HW Whitehead CM Liu L. Exisulind, a novel proapoptotic drug, inhibits rat urinary bladder tumorigenesis. Cancer Res (2001) 61(10):3961–8. 11358813

[104] Pantziarka P Bouche G Sullivan R Ilbawi AM Dare AJ Meheus L. Perioperative therapies – Enhancing the impact of cancer surgery with repurposed drugs. Eur J Surg Oncol (2017) 43(11):1985–88. 10.1016/j.ejso.2017.08.010 28928011

[105] Tai L-H Alkayyal AA Leslie AL Sahi S Bennett S Tanese de Souza C. Phosphodiesterase-5 inhibition reduces postoperative metastatic disease by targeting surgery-induced myeloid derived suppressor cell-dependent inhibition of Natural Killer cell cytotoxicity. Oncoimmunology (2018) 7(6):e1431082–e1431082. 10.1080/2162402X.2018.1431082 29872554 PMC5980420

